# Immunization of Health-Care Providers: Necessity and Public Health Policies

**DOI:** 10.3390/healthcare4030047

**Published:** 2016-08-01

**Authors:** Helena C. Maltezou, Gregory A. Poland

**Affiliations:** 1 Department for Interventions in Health-Care Facilities, Hellenic Center for Disease Control and Prevention, 3-5 Agrafon Street, Athens 15123, Greece; 2 Mayo Clinic Vaccine Research Group, 611C Guggenheim Building, Mayo Clinic and Foundation, 200 First Street, SW Rochester, Rochester, MN 55905, USA; poland.gregory@mayo.edu

**Keywords:** vaccine-preventable diseases, immunization, vaccination, health-care providers, health-care workers, health-care associated, nosocomial, outbreak, occupational risk, policies, mandatory

## Abstract

Health-care providers (HCPs) are at increased risk for exposure to vaccine-preventable diseases (VPDs) in the workplace. The rationale for immunization of HCPs relies on the need to protect them and, indirectly, their patients from health-care-associated VPDs. Published evidence indicates significant immunity gaps for VPDs of HCPs globally. Deficits in knowledge and false perceptions about VPDs and vaccines are the most common barriers for vaccine uptake and may also influence communication about vaccines between HCPs and their patients. Most countries have immunization recommendations for HCPs; however, there are no universal policies and significant heterogeneity exists between countries in terms of vaccines, schedules, frame of implementation (recommendation or mandatory), and target categories of HCPs. Mandatory influenza immunization policies for HCPs have been implemented with high vaccine uptake rates. Stronger recommendations for HCP immunization and commitment at the level of the health-care facility are critical in order to achieve high vaccine coverage rates. Given the importance to health, mandatory immunization policies for VPDs that can cause serious morbidity and mortality to vulnerable patients should be considered.

## 1. Introduction

**The time is always right to do what is right.**

**Martin Luther King, Jr. (1929–1968)**

The widespread implementation of vaccinations during the second half of the past century has had an exceptional impact on childhood morbidity and mortality globally [1]. Outbreaks of vaccine-preventable diseases (VPDs) still challenge health-care facilities, even in countries with well-established immunization programs, resulting in considerable morbidity and fatal events among patients and health-care providers (HCPs) [2,3,4,5,6,7,8,9,10]. Historically, the 1982 recommendations of the United States Advisory Committee on Immunization Practices (ACIP) for HCP immunization against hepatitis B, followed by the 1984 ACIP recommendations for influenza immunization, paved the way for occupational immunization programs for this high-risk occupational group [11]. Currently immunization programs for HCPs constitute a key role in occupational medicine and infection control within health-care facilities, and several countries and health-care facilities have established comprehensive immunization programs for HCPs [12,13,14,15]. In this article, the rationale for immunizations of HCPs, issues of HCP attitudes about and acceptance of occupational immunizations, HCP immunization rates, and immunization policies will be reviewed.

## 2. Rationale for Immunization of HCPs

The rationale for immunization of HCPs relies on two facts: first, susceptible HCPs are at increased risk for occupational acquisition of VPDs. Analysis of past data indicates that HCPs have an increased risk for acquisition of measles [16] and influenza compared to adults working in non-healthcare settings [17]. In the modern vaccine era, as vaccines decrease the incidence of VPDs, the respective average patient age has increased in many developed countries, shifting from the first to the third decade of life; thus, it is expected that the severity of several formerly childhood VPDs will increase [18]. A modeling study showed a 4.5-fold increased risk of adverse outcomes from measles, a 2.2-fold increased risk from varicella, and a 5.8-fold increased risk from rubella in a modern-day scenario relative to the pre-vaccination era [18]. HCP immunization is recommended for protection of older persons, and is especially important for HCPs who have underlying diseases (e.g., immunosuppression, chronic diseases) or specific conditions (pregnancy, elderly); however, this issue is usually not addressed [19].

Second, HCPs provide health care to patients, many of whom are at high risk for a serious disease course, complications, or even death because of their age (e.g., neonates, young infants, elderly) and/or underlying conditions (e.g., pregnant women, immunocompromised patients, patients with underlying diseases). These groups are frequent users of health-care services, yet often they do not develop a strong immune response after immunization [20] or they are ineligible for immunization (e.g., the measles-mumps-rubella (MMR) vaccine is regularly administered after 12 months of age and is contraindicated during pregnancy) [21,22].

HCPs have been traced as the primary source of infection in many outbreaks of VPDs, including influenza, pertussis, measles, rubella, varicella, hepatitis A, and hepatitis B [10,21,22,23,24,25,26,27]. In several pertussis outbreaks in neonatal intensive care units, the diagnosis was not considered early enough; although HCPs had compatible symptoms, the HCPs continued working despite being contagious [21]. A recent study showed that nearly half of 41 HCPs with symptomatic laboratory-confirmed influenza were afebrile, posing a risk of influenza transmission to their patients and colleagues [27]. Beyond their impact on morbidity and mortality, VPD outbreaks in healthcare facilities are also associated with absenteeism among personnel, disruption of health-care services, and extensive costs because of testing, prophylaxis, treatment, infection-control measures, and contact tracing [21,22,23,28]. Therefore, immunization of HCPs is justified in order to prevent transmission of VPDs to susceptible patients, their colleagues, and families; to reduce morbidity and absenteeism among them; and to preserve health-care services [29,30,31].

## 3. Immunity Status of HCPs

Data about HCP immunity against VPDs are largely fragmentary. Recent studies from several countries indicate significant immunity gaps against many VPDs. Susceptibility rates of HCPs range from 4.6% to 17% for measles, 15.7% to 25% for mumps, 4.5% to 18.6% for rubella, 4.1% to 16.7% for varicella, 48.3% to 68.8% for pertussis, 22.6% to 35% for hepatitis B, and 21.2% to 64.3% for tetanus and diphtheria [7,32,33,34,35,36,37,38,39,40,41,42,43,44,45,46]. Completed immunization rates are also suboptimal globally, ranging from 18.8% to 70.5% against measles and mumps, 22.2% to 70.5% against rubella, 1.9% to 3% against varicella, 0% to 49% against pertussis, 3.6% to 5.8% against hepatitis A, 40% to 95% against hepatitis B, and 35.7% to 47.3% against tetanus-diphtheria [34,35,36,37,38,39,43,46,47,48]. Unknown immunization status is also common among HCPs. Despite the fact that annual influenza immunization has been consistently recommended for the past three decades, influenza vaccine uptake by HCPs remains low over the last few years, ranging from 5% to 42% in many countries except the United States [34,48,49,50,51,52,53,54]. Of note, health-care students are a specific sub-group of HCPs, since they share time between hospital wards and universities, which are often overlooked in immunization programs [55]. Several studies show that health-care students have significant immunity gaps and low immunization coverage rates against VPDs, which reflect immunization gaps during childhood or adolescence and the fact that they are not identified as HCPs by occupational medicine services in health-care facilities [55,56,57,58,59].

## 4. Attitudes and Practices of HCPs toward Vaccines

Studies of vaccine attitudes and HCP practices have focused almost exclusively on influenza immunization. The following barriers to increasing influenza vaccine uptake among HCPs have been consistently identified: gaps in knowledge about influenza, misconceptions about their own risk for contracting influenza, vaccine effectiveness, vaccine safety and vaccine adverse events, lack of convenient access to vaccine, unawareness of the recommendations for annual immunization against influenza, fear of injections, lack of leadership support, sense of autonomy, sense of lesser responsibility, and the influence and use of homeopathic medicine [34,48,50,60,61,62,63,64]. Similar knowledge gaps and negative attitudes toward influenza immunization have been recorded in medical students in Germany and the Netherlands, which indicates that education about influenza and vaccine safety is needed early in medical training [65,66]. The following factors have been associated with increased influenza vaccine uptake among HCPs: on-site immunization, free-of-charge immunization, immunization at convenient times and locations, history of previous influenza immunization, older age, education about influenza and influenza vaccine, organization of campaigns, use of mobile immunization teams, implementation of mandatory immunization policies, use of declination forms, mandatory use of masks by unvaccinated HCPs, use of reminding systems, use of incentives, and leadership support [34,51,60,61,67].

## 5. Immunization Policies for HCPs

Although immunization recommendations for HCPs have been in place for more than three decades in many countries, immunization programs for HCPs differ significantly between countries in terms of vaccines, immunization schemes, implementation frames (mandatory or voluntary), and targeted HCP groups [12,13,14,15,67]. For example, measles immunization is mandatory for HCPs in Finland and voluntary for all or specific groups of HCPs in 18 European countries; no immunization policies for HCPs against measles are in place in the remaining 11 European countries [67]. Similarly, immunization against hepatitis B is mandatory in nine European countries and recommended in 20 countries [67]. Immunization against hepatitis B and influenza are almost uniformly recommended in most countries. With the notable exemption of the United States, where mandatory influenza immunization policies have been in place at many medical facilities with nearly 100% vaccine uptake rates [68,69,70,71], mandatory immunizations for HCPs have been implemented rarely and only for specific conditions elsewhere [12,72]. In contrast, voluntary-based immunization programs for HCPs have not succeeded in achieving high and sustainable vaccine coverage rates. Recent studies indicate wide rates of acceptance of mandatory immunization policies for HCPs, which are largely influenced by the specific vaccine, the specific group of HCPs, and the patients under care [34,37,38,39,72,73,74,75,76].

High influenza immunization rates have also been achieved in a few health-care facilities through consistent multimodal strategies without a mandatory policy [77,78,79,80]. Of note, the baseline immunization rates in most of these studies were well above the influenza immunization rates that are recorded globally (<30%). Successful voluntary-based approaches are demanding in terms of human resources, time, and health-care facility commitment [77,78,79,80,81,82]. Sustainability of high vaccine uptake rates is also difficult to achieve [51].

## 6. Authors’ Recommendations

Mandatory immunization policies should be implemented against VPDs that can be transmitted and cause significant morbidity and mortality to patients, and for which safe and effective vaccines exist. Legislative, as well as cultural, issues should be considered in order to strengthen immunization policies for HCPs.

Practical issues of vaccine delivery to HCPs, including the development of in-hospital registries, follow-up procedures, and reminder systems, should also be addressed [67]. Barriers to providing accurate immunization coverage data are expected to decrease over time as facilities become familiar with reporting [83]. Lastly, behavior and education are increasingly recognized as core determinants of HCP attitudes and practices toward immunizations [84,85]. Communication is critical in order to overcome HCP concerns and mistrust of vaccines and raise immunization rates. Medical schools and advanced training programs (residency, fellowship) should incorporate comprehensive education about vaccines in their curricula [67]. HCPs at all levels have a professional and moral responsibility to protect the patients they are privileged to care for; professional responsibility and ethics should be addressed at the university level and through professional societies, especially if mandatory immunization policies are adopted [86,87].

## 7. Conclusions

Data from the past three decades consistently indicate that voluntary immunization policies for HCPs have not achieved and sustained high immunization rates. Health-care facility commitment is required in order to eliminate HCP misconceptions about VPDs and vaccines and raise immunization rates, especially if mandatory immunization policies are widely implemented. The issue of mandatory immunizations should be considered for VPDs that can cause significant morbidity and mortality to patients, in order to induce immunity and promote safety both at the HCP level and at the level of the health-care facility (herd immunity).

## Abbreviations

The following abbreviations are used in this manuscript:

HCPs	health-care providers
VPDs	vaccine-preventable diseases
ACIP	Advisory Committee on Immunization Practices
MMR	measles-mumps-rubella
